# Bioconversion variation of ginsenoside CK mediated by human gut microbiota from healthy volunteers and colorectal cancer patients

**DOI:** 10.1186/s13020-021-00436-z

**Published:** 2021-03-17

**Authors:** Yin-Ping Guo, Li Shao, Li Wang, Man-Yun Chen, Wei Zhang, Wei-Hua Huang

**Affiliations:** 1 Department of Clinical Pharmacology, Xiangya Hospital, Central South University, Changsha, 410008 China; 2 Department of Pharmacognosy, School of Pharmacy, Hunan University of Chinese Medicine, Changsha, 410128 Hunan China; 3 Institute of Clinical Pharmacology, Hunan Key Laboratory of Pharmacogenetics, Central South University, Changsha, 410078 China; 4 National Clinical Research Center for Geriatric Disorders, Xiangya Hospital, Central South University, Xiangya Road 110, 410008 Changsha, China; 5 NHC Key Laboratory of Birth Defect for Research and Prevention (Hunan Provincial Maternal and Child Health Care Hospital), Changsha, 410008 Hunan China

**Keywords:** Ginsenoside CK, Gut microbiota, LC-MS/MS, Colorectal cancer, 16S rRNA gene sequencing

## Abstract

**Background:**

Ginsenoside CK (GCK) serves as the potential anti-colorectal cancer (CRC) protopanaxadiol (PPD)-type saponin, which could be mainly bio-converted to yield PPD by gut microbiota. Meanwhile, the anti-CRC effects of GCK could be altered by gut microbiota due to their different diversity in CRC patients. We aimed to investigate the bioconversion variation of GCK mediated by gut microbiota from CRC patients by comparing with healthy subjects.

**Methods:**

Gut microbiota profiled by 16S rRNA gene sequencing were collected from healthy volunteers and CRC patients. GCK was incubated with gut microbiota in vitro. A LC-MS/MS method was validated to quantify GCK and PPD after incubation at different time points.

**Results:**

The bioconversion of GCK in healthy subjects group was much faster than CRC group, as well as the yield of PPD. Moreover, significant differences of PPD concentration between healthy subjects group and CRC group could be observed at 12 h, 48 h and 72 h check points. According to 16S rRNA sequencing, the profiles of gut microbiota derived from healthy volunteers and CRC patients significantly varied, in which 12 differentially abundant taxon were found, such as *Bifidobacterium*, *Roseburia*, *Bacteroides* and *Collinsella*. Spearman’s correlation analysis showed bacteria enriched in healthy subjects group were positively associated with the biotransformation of GCK, while bacteria enriched in CRC group displayed non correlation character. Among them, *Roseburia* which could secrete *β*-glycosidase showed the strongest positive association with the bioconversion of GCK.

**Conclusions:**

The bioconversion of GCK in healthy subjects was much faster than CRC patients mediated by gut microbiota, which might alter the anti-CRC effects of GCK. 
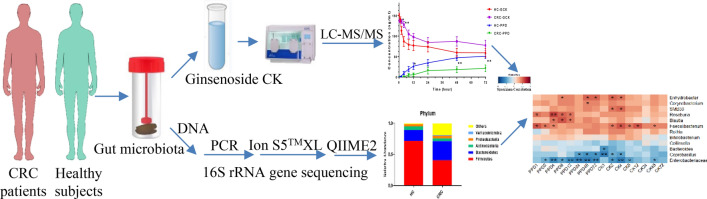

**Supplementary Information:**

The online version contains supplementary material available at 10.1186/s13020-021-00436-z.

## Introduction

Ginsenoside CK (GCK) is one of the most abundant metabolites of protopanaxadiol (PPD)-type saponins bio-converted by gut microbiota in the intestinal tract, which is the major adscription plasma substance of PPD-type saponins into circulatory system [1, 2]. GCK exhibits various pharmacological properties, i.e., anti-colorectal cancer (CRC) and anti-inflammation effect [3, 4]. Interestingly, GCK shows stronger anti-CRC effects than its parent ginsenosides, such as ginsenoside Rb_1_, Rc and Rd [5, 6]. Meanwhile, GCK could be metabolized by gut microbiota to generate the aglycon PPD, which could also be determined in plasma after oral administration of GCK in phase I clinical trial [7, 8]. The absolute oral bioavailability of GCK is about 35% in rats [9], while the key enzyme for catalyzing GCK to yield PPD is *β*-glycosidase, which could only be secreted by gut microbiota, not human beings [10]. The pertinent results have indicated that GCK could be delivered into intestinal tract and further metabolized by gut microbiota after oral administration [11]. Moreover, PPD could also inhibit the proliferation of HCT-116 cells and the growth of xenograft tumor in athymic nude mice [12–14]. The data imply that the bioconversion of GCK mediated by gut microbiota could lead to different responses on its anti-CRC effects.

Gut microbiota could be modulated by many factors such as physiologic health and disease including CRC [15, 16]. The profiles of gut microbiota are significantly different between CRC patients and healthy volunteers [17, 18]. For example, *Bifidobacterium* and *Lactobacillus* are significantly higher in healthy volunteers than CRC patients, while healthy volunteers possess a relative lower abundance of *Bacteroides*. Studies have verified that *Bifidobacterium* and *Lactobacillus* could secret some enzymes to hydrolyze the glycosidic bond, such as *β*-glycosidase [19, 20]. Herein, the bioconversion of GCK might be very different due to the variation of gut microbiota between healthy subjects and CRC patients, which could alter the anti-CRC effects of GCK. Therefore, it is meaningful to investigate the bioconversion of GCK mediated by gut microbiota derived from CRC patients and healthy volunteers.

In this study, GCK and PPD were quantified by a validated LC-MS/MS method after in vitro incubation with gut microbiota at different time points. Gut microbiota collected from CRC patients and healthy volunteers were profiled by 16S rRNA gene sequencing. The results indicated that the bioconversion of GCK mediated by gut microbiota derived from healthy subjects was much faster than CRC patients.

## Materials and methods

### Chemical and materials

**Fig. 1:**
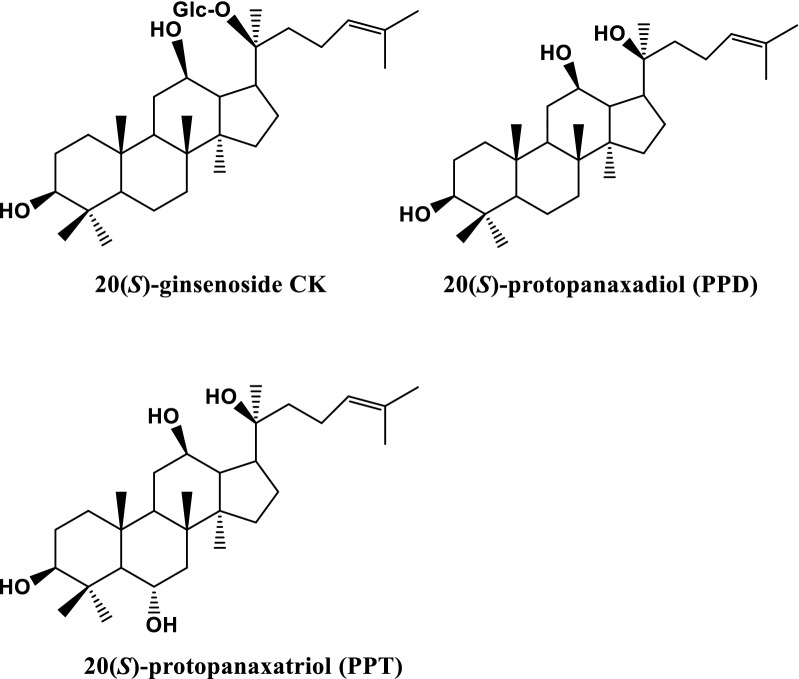
Chemical structure of GCK, PPD and PPT

The standards, 20(*S*)-GCK and 20(*S*)-protopanaxatriol (PPT), were purchased from Chengdu Push Bio-technology Co., Ltd (Sichuan, China), and 20(*S*)-PPD was supplied by Baoji Herbest Bio-Tech Co., Ltd (Shaanxi, China). Their chemical structures were presented in Fig. 1. Acetonitrile (ACN) and methanol in HPLC-grade were obtained from Merck Company (Darmstadt, Germany).

### Fecal samples preparation

The stool samples were collected from 11 CRC patients (CRC group) and 11 healthy volunteers (health group), respectively, who had not taken any probiotics or antibiotics in the last 30 days and abused alcohol or tobacco. CRC patients were screened and diagnosed through physical examination, who had similar body-mass index value with healthy volunteers (22.69 ± 1.69 for CRC group, and 21.58 ± 1.72 for health group, Additional file 1: Table S1). Gut microbiota were prepared from fresh fecal samples immediately, while the remained samples were stored in a − 80 ℃ freezer until sequencing.

### Biotransformation of GCK

Fresh fecal samples (1 g) were suspended in 15 mL of cold sterile physiological saline by following the preparation process described in our previous work [11]. The stocks of gut microbiota suspension were cultured with 10 fold volume of general anaerobic medium (GAM) broth in an E500 anaerobic chamber (Gene Science, USA) at 37 ℃ for activation. After incubation for 24 h, the suspension was centrifuged at 4000 rpm for 20 min at 4 ℃, and then 5 mL of GAM broth was used to re-suspend the precipitate as work solution of gut microbiota.

The biotransformation of GCK mediated by gut microbiota in vitro was performed in 0.5 mL of incubation system, which contained 2.5 µL of GCK solution (dissolved in DMSO, 50 µg/mL), 100 µL of work solution of gut microbiota and 398 µL of GAM broth. The mixture was incubated at 37 ℃ in the anaerobic chamber (80% N_2_, 10% H_2_ and 10% CO_2_) for 1 h, 2 h, 4 h, 8 h, 12 h, 24 h, 48 h and 72 h. Samples were prepared in triplicate for each time check point.

### Sample preparation

The incubation mixture was extracted with 0.5 mL of ethyl acetate and 0.5 mL of water saturated *n*-butanol by using a vortex for 10 min, respectively. The extracted supernatant was mixed together and dried under nitrogen at room temperature. The residues were re-dissolved with 100 µL of methanol and then centrifuged at 13,000 rpm for 10 min before analysis.

### Instrumentation and analytical conditions

GCK and PPD were quantified in the negative ion mode on an AB SCIEX 6500 + Triple Quad LC-MS/MS system (AB SCIEX, UAS). The chromatographic separation was achieved on an ACQUITY BEH Shield RP18 column (50 × 2.1 mm, 1.7 μm, Waters, USA) with a gradient elution of 2 mM ammonium acetate in water (A) and ACN (B) at a flow rate of 0.3 mL/min. The gradient profile was optimized as following, 20% (B) for 0.01–1 min, 20−65% (B) for 1–2 min, 65–75% (B) for 2–3 min, 75–90% (B) for 3–4 min, 90–100% (B) for 4–5 min and 100% (B) for 5–6 min. The mass spectrometer parameters were optimized as following, ion source gas 1 and 2, 45 psi (nitrogen); curtain gas, 25 psi (nitrogen); temperature, 450 ℃ and ion spray voltage, − 4500 V. The optimized MRM parameters of each compound were listed in Additional file 1: Table S2. The injection volume was 2 µL, while the system was controlled by AB SCIEX Analyst TF software (version 1.6).

### Calibration standards and quality control (QC) samples

The primary stock solutions of GCK (0.408 mg/mL), PPD (0.402 mg/mL) and PPT (IS, 0.196 mg/mL) were prepared by dissolving each compound in methanol, respectively. Working solutions were prepared by diluting the stock solutions appropriately with methanol-water (V:V = 1:1) and stored at 4 ℃. The standard calibration curves samples and QC samples were prepared by spiking 2.5 µL of working solution with 100 µL of gut microbiota work solution and 398 µL of GAM broth. The solution of PPT (IS) was diluted to 1.96 µg/mL using methanol-water.

### Method validation

The method was validated by following respect to selectivity, sensitivity, linearity, precision, accuracy, recovery and matrix effect according to the United States Food and Drug Administration (FDA) guidelines. The selectivity of this method was evaluated by comparing chromatograms of extracted blank gut microbiota solution obtained from six different human feces samples with those spiked with GCK (0.51 µg/mL), PPD (0.5025 µg/mL) and PPT (1.96 µg/mL) work solutions. The seven-point standard curves, ranging from 2.55 to 408 ng/mL for GCK and 2.51 to 402 ng/mL for PPD, were plotted on the peak area ratio of target ions to the IS versus the corresponding concentrations by a weighted (1/X^2^) linear least squares regression model. The intra- and inter-day precision and accuracy were determined by analyzing six replicate samples at each QC level within one day and three consecutive days. The QC samples were set at 2.55 ng/mL, 7.65 ng/mL, 51 ng/mL and 306 ng/mL for GCK, and 2.51 ng/mL, 7.54 ng/mL, 50.25 ng/mL and 301.5 ng/mL for PPD. The recovery and matrix effect were investigated accordingly.

### 16S rRNA gene sequencing

Microbial genomic DNA was extracted from fecal samples by using Qiagen QIAamp DNA Stool Mini Kit (Qiagen, Germany), and the V3-V4 region of the bacteria 16S rRNA gene was amplified by PCR using primers 341F 5′-CCTAYGGGRBGCASCAG-3′ and 806R 5′-GGACTACNNGGGTATCTAAT-3′. Amplicons were extracted and purified by using Gene JET Gel Extraction Kit (ThermoScientific, USA). Sequencing libraries were constructed by using Ion Plus Fragment Library Kit 48 rxns (ThermoFisher, USA). Purified amplicons were sequenced on Ion S5^TM^XL platform (ThermoFisher, USA) according to the standard protocols.

### Bioinformatics analysis

Bioinformatics analysis of bacteria 16S rRNA sequencing data was conducted by using QIIME 2 software [21, 22] (version 2018.11, https://qiime2.org). Raw sequence data were developed into clean data by cut off the barcode and primer sequences (Cutadapt plugin), de-multiplexed (demux plugin), and denoised with DADA2 (Dada2 plugin) [23]. Operational Taxonomic Units (OTUs) were clustered with 97% similarity cutoff, and the GreenGene Database (http://greengenes.lbl.gov/cgi-bin/nph-index.cgi) was applied for the taxonomic classification. Alpha diversity and beta diversity were analyzed by Diversity plugin.

To analyze the alpha diversity, Shannon index, Observed OTUs, Faith’s index and Pielou’s index were calculated to assess community diversity, richness, phylogenetic diversity and evenness, respectively. The data were visualized by using GraphPad Prism (version 7.00). Beta diversity was assessed by using principal co-ordinates analysis (PCoA). Bray Curtis distance and Jaccard distance were calculated according to phylogenetic measures, while Unweighted Unifrac distance and Weighted Unifrac distance were employed for the sequence measures. Moreover, the linear discriminative analysis effect size (LDA effect size, LEfSe) analysis was conducted to discover the differentially abundant taxon by using LEfSe software (LDA score > 4). Furthermore, Phylogenetic Investigation of Communities by Reconstruction of Unobserved States (PICRUSt) analysis was used to predict the metabolic function of bacteria, of which the different function was expressed as LDA scores (LDA scores > 2). Lastly, a heat map of Spearman’s correlations was constructed by using gplots and RColorBrewer packages of R software (version 4.0.0).

### Statistical analysis

Statistical analyses was performed on SPSS software (version 17.0), and student’s *t* test was used to evaluate the biotransformation and alpha diversity. Kruskal-Wallis test and Wilcoxon test were employed to assess LEfSe and the relative abundance of bacteria. Significant differences were expressed as *p < 0.05, **p < 0.01 and #*p* < 0.001.

## Results

### Method validation

The developed method displayed a good selectivity for GCK and PPD (Additional file 2: Fig. S1). Both calibration curves (Additional file 1: Table S3) showed good linearity (r ≥ 0.9987), and the LLOQ was 2.55 ng/mL for GCK and 2.51 ng/mL for PPD. The intra- and inter-day precision (Additional file 1: Table S4) was ranged from 1.8 to 7.2% for GCK and 2.1–9.8% for PPD, while the intra- and inter-day accuracy (Additional file 1: Table S4) was ranged from 95.5 to 100.1 % for GCK and 94.7–102.5% for PPD. Additional file 1: Table S5 showed the results of recovery and matrix effect. The recovery of GCK and PPD was ranged from 86.3 to 92.9  and 85.8–94.0%, respectively. The matrix effect of GCK and PPD were ranged from 4.6 to 8.3% and 6.7–8.9%, respectively, which indicated that no endogenous substances significantly suppressed or enhanced the ionization of both compounds, as well as the IS. Overall, the developed LC-MS/MS method was successfully validated to quantify GCK and PPD in gut microbiota incubation system.

### Biotransformation of GCK

**Fig. 2:**
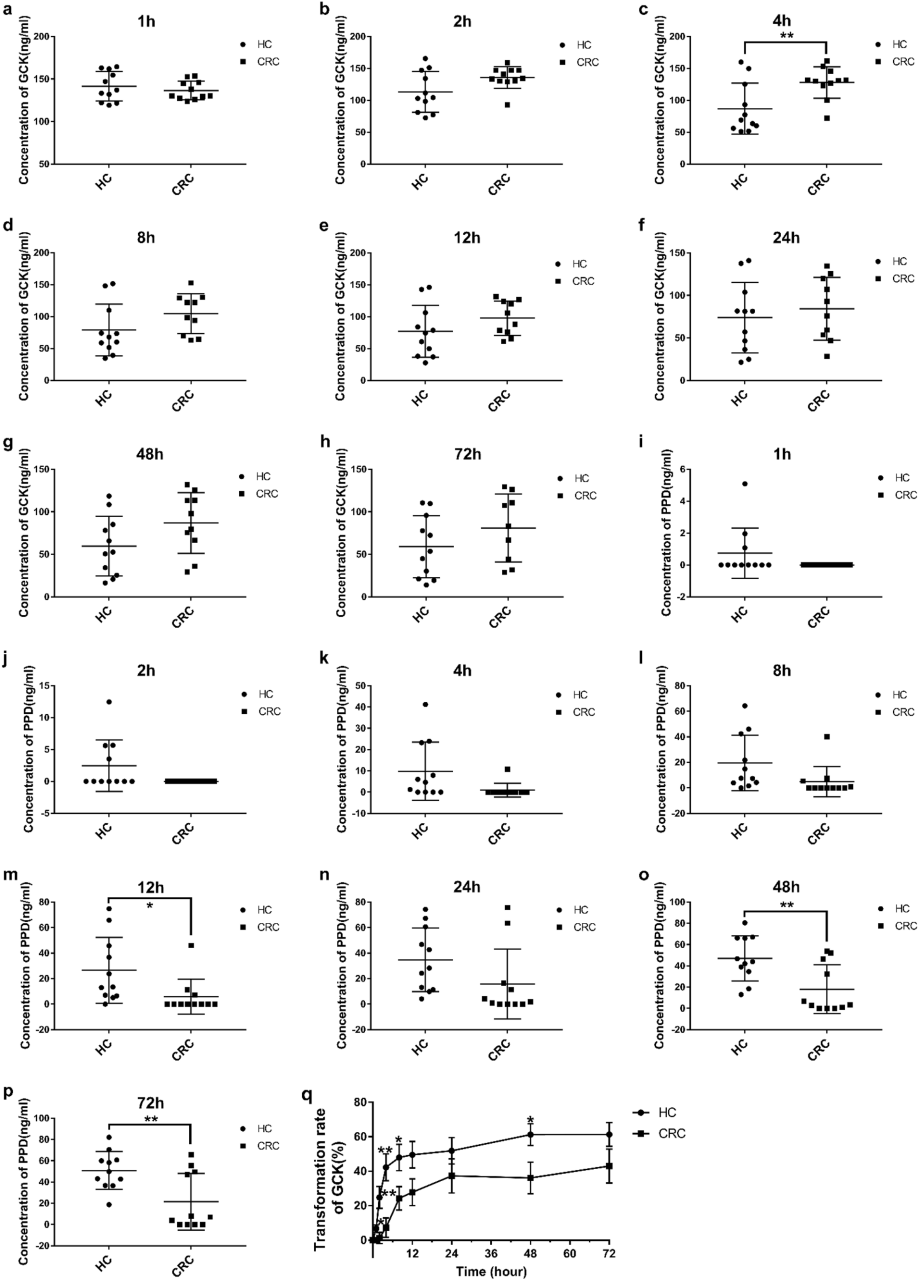
Quantitative analysis of GCK by gut microbiota. **a**–**h** mean concentration of GCK in the incubation system among 1–72 h between health and CRC group; **i**–**p** mean concentration of PPD among 1–72 h; **q** bioconversion rate of GCK (data were expressed as mean ± SEM); *p < 0.05 and **p < 0.01; HC: health group; CRC: CRC group

As shown in Table 1; Fig. 2a–p, compared with CRC group, the concentration of GCK in the health group decreased rapidly during 0 h to 4 h. PPD could be initially detected at 1 h in the health group, but at 4 h in the CRC group, approximately. Meanwhile, the concentration of PPD at 12 h, 48 h and 72 h in the health group was significantly higher than CRC group. The bioconversion rate of GCK (Fig. 2q) in the health group was much higher than CRC group at 1–8 h and 48 h. The bioconversion variation of GCK was unambiguously observed between health group and CRC group, which implied gut microbiota profiles were significantly different between health group and CRC group.

**Table 1:** Mean concentration of GCK and PPD in the incubation system at each time point

Time (h)	Health Group (ng/mL)		CRC group (ng/mL)	
	GCK	PPD	GCK	PPD
0	150.26	0	137.82	0
1	141.50	0.74	136.54	0
2	113.33	2.48	135.62	0
4	86.99	9.85	127.88	0.98
8	79.08	19.48	104.60	4.90
12	76.90	26.57	97.75	5.85
24	73.94	34.80	84.43	15.87
48	59.70	47.06	86.93	18.05
72	56.06	50.90	77.28	21.51

### Alpha and beta diversity of gut microbiota

**Fig. 3:**
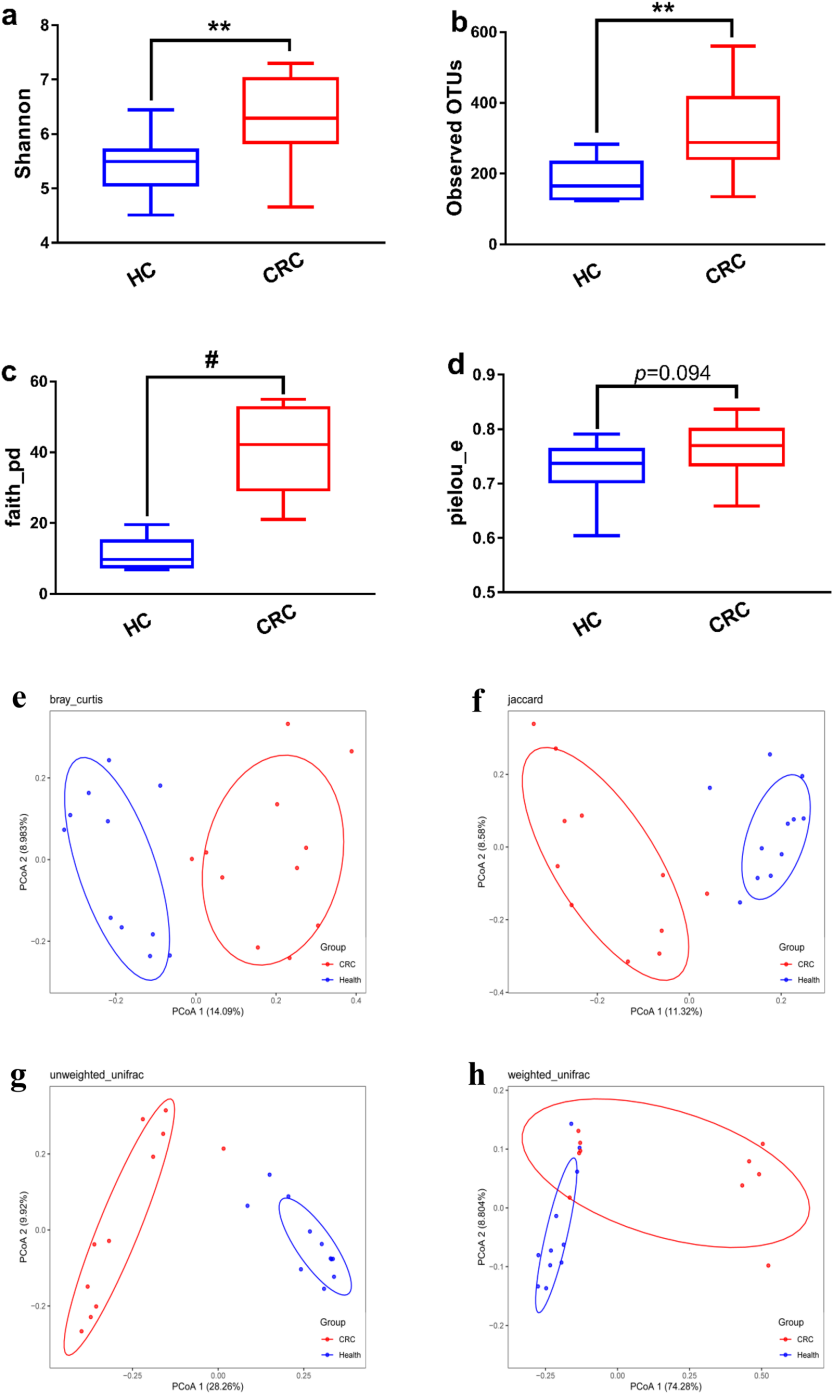
Alpha diversity (**a**–**d**) and beta diversity (**e**–**h**) of gut microbiota. **a** Shannon index; **b** Observed OTUs; **c** Faith’s index; **d** Pielou’s index; **e** Bray Curtis distance; **f** Jaccard distance; **g** unweighted Unifrac distance; **h** weighted Unifrac distance; HC: health group; CRC: CRC group

As presented in Fig. 3a–d Shannon index, Observed OTUs and Faith’s index had higher levels in CRC group than health group, which implied a higher alpha diversity within microbial communities of CRC group. Beta diversity (Fig. 3e–

h) showed that all samples could be unambiguously clustered into two groups. The 16S rRNA sequencing data provided that the alpha and beta diversity of gut microbiota were significantly different between health group and CRC group.

### Taxonomic differences of gut microbiota

**Fig. 4:**
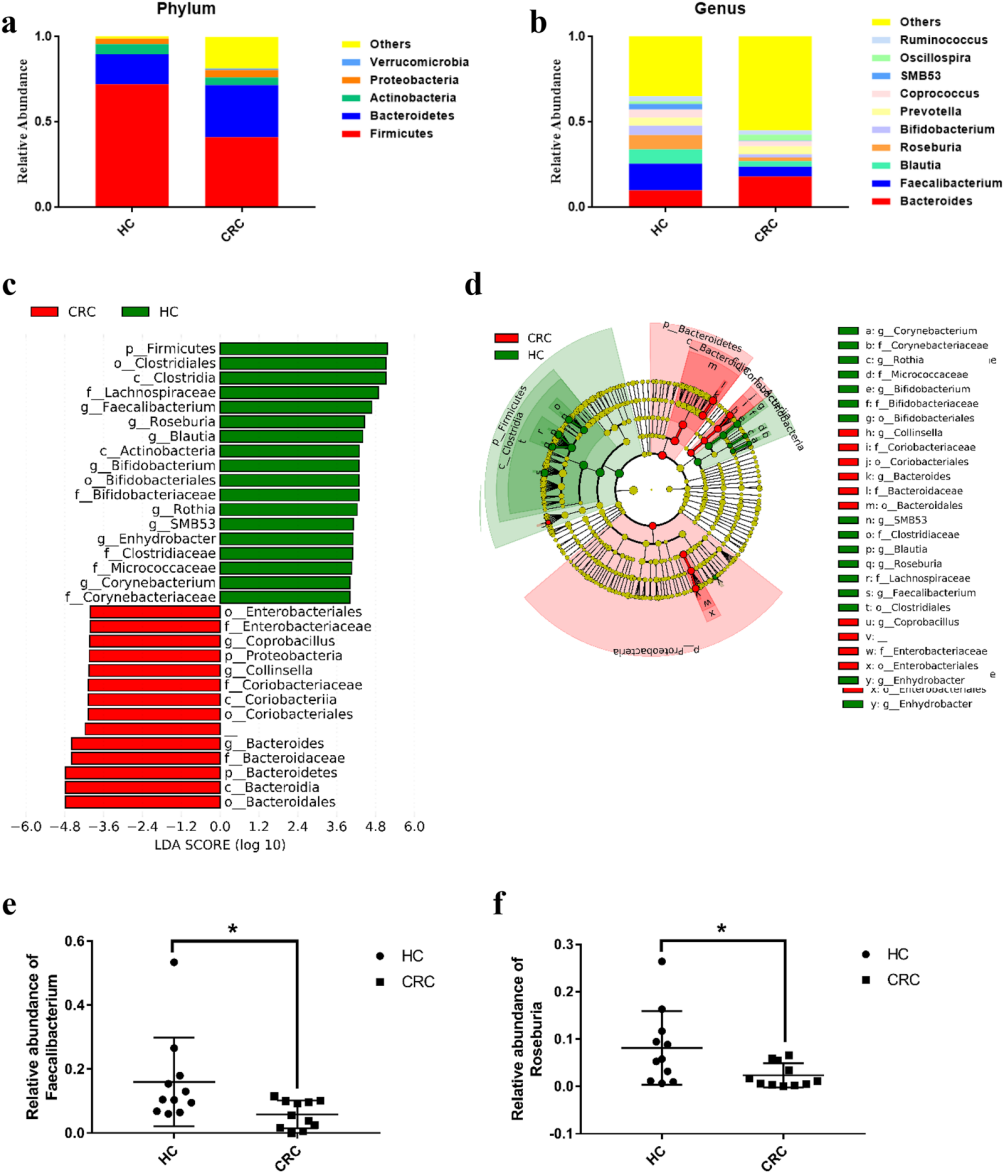
Bacteria annotation in phylum level and genus level (**a**–**b**) and LEfSe analysis (**c**–**f**). **a** Relative abundance of top five bacteria in the phylum level between two groups; **b** Relative abundance of top ten bacteria in the genus level; **c** gut microbiota observed between health and CRC group (*p* < 0.05, LDA scores > 4); **d** Cladogram based on LDA scores; **e** Relative abundance of *Faecalibacterium* in each sample; **f** Relative abundance of *Roseburia*; significant difference of relative abundance were analyzed using Kruskal-Wallis test and Wilcoxon test; “_” means unclassified bacteria; HC: health group; CRC: CRC group

Taxonomical classification was performed on five levels (phylum, class, order, family and genus). Compared with CRC group, the relative abundance of *Firmicutes* was much higher in health group, while *Bacteroidetes* significantly decreased (Fig. 4a). Top ten bacteria of both groups in genus level (Fig. 4b) were *Bacteroides*, *Blautia*, *Bifidobacterium*, *Coprococcus*, *Faecalibacterium*, *Oscillospira*, *Prevotella*, *Roseburia*, *Ruminococcus* and *SMB53*.

We also used LEfSe analysis to discover the differentially abundant taxon. In genus level, 12 bacteria (*p* < 0.05, LDA scores > 4) were found, which could be mainly categorized into *Actinobacteria*, *Bacteroidetes*, *Firmicutes* and *Proteobacteria* (Phylum) (Additional file 1: Table S6). Figure 4c–d showed that *Bifidobacterium*, *Blautia*, *Corynebacterium*, *Enhydrobacter*, *Faecalibacterium*, *Roseburia*, *Rothia* and *SMB53* were enriched in health group, while *Bacteroides*, *Collinsella*, *Coprobacillus* and *Enterobacteriaceae* increased comparatively in CRC group. Among them, *Bacteroides*, *Bifidobacterium*, *Blautia*, *Faecalibacterium*, *Roseburia*, and *SMB53* were the predominant abundant bacteria in genus level (Fig. 4e, f and Additional file 2: Fig. S2).

Lastly, the metabolic function of bacteria was predicted using PICRUSt analysis. A total of 328 KEGG pathways had been enriched, 41 functional pathways of which showed significant differences between health group and CRC group (*p* < 0.05, LDA scores > 2). The data (Additional file 2: Fig. S3) showed that ABC transporters, sporulation, porphyrin and chlorophyll metabolism were obviously enriched in health group, while amino and nucleotides metabolism and lipopolysaccharide biosynthesis proteins were enriched in CRC group. These findings proved that the profiles and functions of gut microbiota derived from CRC group were discriminated from health group.

### Correlation between the biotransformation of GCK and gut microbiota

**Fig. 5:**
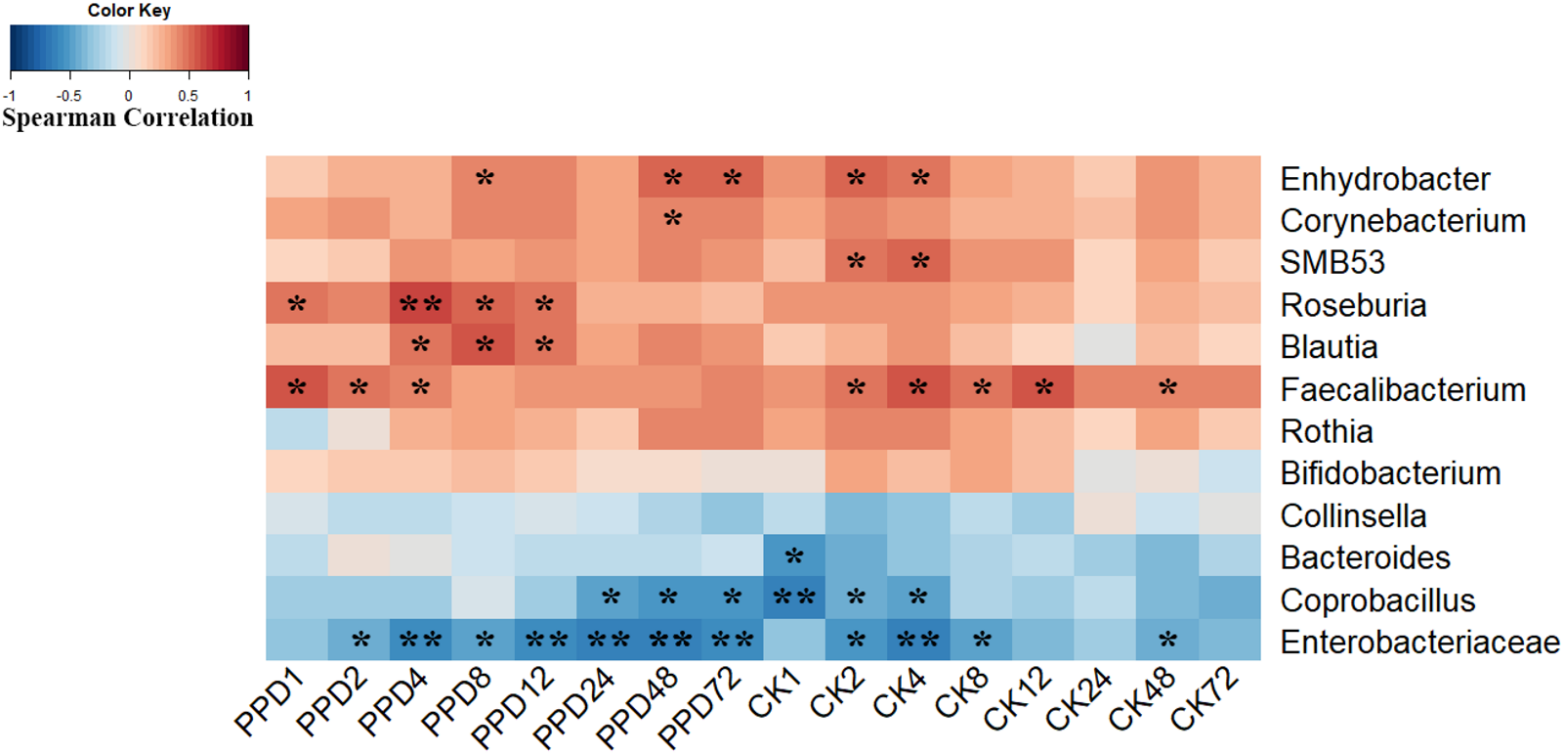
Heatmap of Spearman’s correlations between biotransformation of GCK and 12 differentially abundant taxon. CK 1–72 means bioconversion rate of GCK with 1–72 h incubation; PPD 1–72 means the concentration of PPD with 1–72 h incubation; * *p* < 0.05 and ** *p* < 0.01

For better understanding the relationship of gut microbiota and bioconversion of GCK, Spearman’s correlation index was calculated between the bioconversion rate of GCK and the relative abundance of 12 differentially abundant taxon (Fig. 5). Bacteria enriched in health group were positively correlated with the biotransformation of GCK, while bacteria enriched in CRC group showed non correlation character. Among them, *Blautia*, *Enhydrobacter*, *Faecalibacterium* and *Roseburia* showed strong positive correlations with biotransformation rate of GCK and PPD yield, while *Coprobacillus* and *Enterobacteriaceae* were negatively correlated with them. In addition, *Roseburia* showed significant correlation with PPD yield after incubation at 1 h, 4 h, 8 h and 12 h, while the Spearman correlation provided the highest value (0.603) between its relative abundance and PPD yield at 4 h. The data implied that *Roseburia* might be a main contributor for the bioconversion variation of GCK between health group and CRC group.

## Discussion

In our study, the bioconversion rate of GCK in the healthy volunteers was higher than CRC patients mediated by gut microbiota, which implied that GCK and PPD absorbed into human plasma would be different between healthy subjects and CRC patients after oral administration with equivalent dosage of GCK. Similarly, *in vivo* effects after oral administration with GCK might also vary. Moreover, PPD also showed different pharmaceutical activities, such as anti-inflammatory and anti-CRC effects [24]. However, anti-CRC effects of GCK pertinent to gut microbiota were still unknown.

In addition, besides genetic impacts, gut microbiota are also re-shaped by many environmental factors, such as gastro-intestinal diseases [25]. Hence, we recruited CRC patients from physical examination center without any drug treatments. Meanwhile, gut microbiota of CRC patients and healthy volunteers were profiled by 16S rRNA gene sequencing due to individual variation. The annotation results showed the ratio of *Firmicutes* phyla to *Bacteroidetes* phyla was lower in CRC subjects, which was consistent with the reported literatures, as well as some top ten gut microbials in genus level, such as *Bacteroides* and *Roseburia* [17]. The results of LEfSe analysis found that *Bifidobacterium* and *Roseburia* were enriched in health volunteers, while *Bacteroides* and *Collinsella* were enriched in CRC patients [17, 26]. These data indicated that profiles of gut microbiota derived from CRC patients and healthy subjects were significantly different.

In addition, the results of Spearman’s correlation analysis found that 4 genus bacteria were positively associated with the bioconversion of GCK. As the metabolism of GCK was catalyzed by *β*-glycosidase, the ability of gut microbiota to secret these key enzymes should be predominantly considered. Studies verifies that more than half of the low G+ C% Gram-positive *Firmicutes* harbored *β*-glycosidase, as well as *Bifidobacterium spp.* and *Lactobacillus* [19, 20]. Meanwhile, *Bifidobacterium*, *Rumnococcus* and *Roseburia* could secret *β*-glycosidase [20, 27]. Importantly, our previous work had also verified that PPD-type ginsenosides could be bio-converted by *Bacteroides ovatus*. Therefore, *Roseburia*, which belongs to butyrate-producing bacteria and alleviates experimental colitis pathology by inducing anti-inflammatory responses (such as *Roseburia intestinalis*) [28, 29], might play an essential role on the bioconversion variation of GCK between healthy subjects and CRC patients.

As gut microbiota provide crucial signals to host immune system, more and more studies have been reporting that drugs have interaction with gut microbiota [24, 30]. In this study, we only focused on the bioconversion of GCK mediated by gut microbiota, but the effects of GCK on gut microbiota were not investigated, which might also play important role on its biological activities.

## Conclusions

A LC-MS/MS method was validated to quantify GCK and PPD in the gut microbiota incubation system. The bioconversion rate of GCK mediated by gut microbiota derived from healthy volunteers was much higher than CRC patients. The profiles of gut microbiota between health subjects and CRC patients were significantly different through 16S rRNA sequencing. *Roseburia* might be a main contributor for bioconversion variation of GCK.

##  Supplementary Information

**Additional file 1: Table S1**. Population Characteristics of recruited volunteers. **Table S2**. MRM parameters of GCK, PPD and PPT. **Table S3**. Linearity range, correlation coefficients (r), calibration curves and LLOQ of GCK and PPD. **Table S4**. Precision and accuracy of GCK and PPD. **Table S5**. Recovery and matrix effect of GCK and PPD. **Table S6**. Taxonomical information of the differentially abundance bacteria by LEfSe analysis.

**Additional file 2: Figure S1.** Representative MRM chromatograms of blank gut microbiota solution, GCK, PPD and PPT. a_1_,b_1_,c_1_ were the corresponding chromatograms of blank gut microbiota solution; a_2_: GCK; b_2_: PPD; c_2_: PPT. **Figure S2**. Relative abundance of differentially abundant bacteria in each sample. (a) Bifidobacterium; (b) Blautia; (c) Corynebacterium; (d) Enhydrobacter; (e) Rothia; (f) SMB53; (g) Bacteroides; (h) Collinsella; (i) Coprobacillus; (j) Enterobacteriaceae; significant difference of relative abundance were analyzed using Kruskal-Wallis test and Wilcoxon test; * *p* < 0.05, ** *p* < 0.01 and # *p* < 0.001. **Figure S3**. Significant functional pathways between health and CRC group by PICRUSt analysis (*p* < 0.05, LDA scores > 2).

## Data Availability

The research data generated from this study are included within the article and supplementary files.

## Abbreviations

ACN: Acetonitrile
GCK: Ginenoside CK
CRC: Colorectal cancer
GAM: General anaerobic medium
LDA: The linear discriminative analysis
LEfSe: The linear discriminative analysis effect size
OTUs: Operational Taxonomic Units
PCoA: Principal co-ordinates analysis
PICRUSt: Phylogenetic Investigation of Communities by Reconstruction of Unobserved States
PPD: Protopanaxadiol
PPT: Protopanaxatriol
QC: Quality control
