# Supplementary material for: Bioconversion variation of ginsenoside CK mediated by human gut microbiota from healthy volunteers and colorectal cancer patients
Source: Chin Med. 2021 Mar 17;16:28. doi: 10.1186/s13020-021-00436-z (PMC7968294; doi:10.1186/s13020-021-00436-z)
Supplement: Supplementary file 1 — Additional file 1: Table S1. Population Characteristics of recruited volunteers. Table S2. MRM parameters of GCK, PPD and PPT. Table S3. Linearity range, correlation coefficients (r), calibration curves and LLOQ of GCK and PPD. Table S4. Precision and accuracy of GCK and PPD. Table S5. Recovery and matrix effect of GCK and PPD. Table S6. Taxonomical information of the differentially abundance bacteria by LEfSe analysis. [file 13020_2021_436_MOESM1_ESM.docx]

**Supplementary Table**

**Bioconversion variation of ginsenoside CK mediated by human gut microbiota from healthy volunteers and colorectal cancer patients**

Supplementary table 1 Population Characteristics of recruited volunteers

| characteristics | CRC volunteers (n=11) | Healthy volunteers (n=11) |
| --- | --- | --- |
| Gender, n (%)  Female | 2(18.18%) | 7(58.33%) |
| Age (years) | 62.73±8.10 | 25.08±2.19 |
| BMI | 22.69±1.69 | 21.58±1.72 |

Supplementary table 2 MRM parameters of GCK, PPD and PPT

| Compounds | Q1/Q3(Da) | Dwell Time(ms) | DP(V) | CE(V) | CXP(V) | EP(V) |
| --- | --- | --- | --- | --- | --- | --- |
| GCK | 621.3/459.5 | 150 | -200 | -26 | -11 | -10 |
| PPD | 459.2/375.5 | 150 | -150 | -39 | -39 | -10 |
| PPT | 475.5/391.5 | 150 | -170 | -40 | -10 | -10 |

Supplementary table 3 Linearity range, correlation coefficients (r), calibration curves and LLOQ of GCK and PPD

| **Compounds** | **Linear range (ng/ml)** | **Correlation coefficients (r)** | **Calibration curves** | **LLOQ (ng/ml)** |
| --- | --- | --- | --- | --- |
| **GCK** | 2.55-408 | 0.9989 | y=0.0443x-0.00264 | 2.55 |
| **PPD** | 2.51-402 | 0.9987 | y=0.0386x-0.00614 | 2.51 |

Supplementary table 4 Precision and accuracy of GCK and PPD

| **Compounds** | **Conc. added (ng/mL)** | **Intra-day (n = 6)** | | **Inter-day (n=18)** | |
| --- | --- | --- | --- | --- | --- |
|  |  | **Accuracy (%)** | **Precision (%)** | **Accuracy (%)** | **Precision (%)** |
| **GCK** | 2.55 | 98.37 | 5.98 | 96.88 | 7.20 |
|  | 7.65 | 97.76 | 1.73 | 99.51 | 2.69 |
|  | 51 | 95.49 | 4.22 | 95.70 | 3.99 |
|  | 306 | 96.46 | 2.62 | 100.1 | 5.89 |
|  |  |  |  |  |  |
| **PPD** | 2.51 | 98.87 | 7.35 | 102.5 | 7.60 |
|  | 7.54 | 97.30 | 2.04 | 101.5 | 9.76 |
|  | 50.3 | 94.69 | 4.72 | 97.42 | 7.43 |
|  | 302 | 98.73 | 5.21 | 99.45 | 5.42 |

Supplementary table 5 Recovery and matrix effect of GCK and PPD

| **Compounds** | **Conc. added**  **(ng/mL)** | **Recovery (%) (n= 6)** | | **Matrix effect (%) (n = 18)** |
| --- | --- | --- | --- | --- |
|  |  | **Mean** | **RSD** | **RSD** |
| **GCK** | 7.65 | 92.90 | 3.70 | 4.51 |
|  | 51 | 89.35 |  | 7.94 |
|  | 306 | 86.31 |  | 8.30 |
|  |  |  |  |  |
| **PPD** | 7.54 | 94.00 | 4.75 | 6.65 |
|  | 50.3 | 92.00 |  | 8.84 |
|  | 302 | 85.77 |  | 8.68 |
| **PPT** | 9.8 | 83.46 | 4.63 | 6.97 |

Supplementary table 6 Taxonomical information of the differentially abundance bacteria by LEfSe analysis

| No./Taxonomy | Kingdom | Phylum | Class | Order | Family | Genus |
| --- | --- | --- | --- | --- | --- | --- |
| 1 | *Bacteria* | *Actinobacteria* | *Actinobacteria* | *Bifidobacteriales* | *Bifidobacteriaceae* | *Bifidobacterium* |
| 2 | *Bacteria* | *Firmicutes* | *Clostridia* | *Clostridiales* | *Lachnospiraceae* | *Blautia* |
| 3 | *Bacteria* | *Actinobacteria* | *Actinobacteria* | *Actinomycetales* | *Corynebacteriaceae* | *Corynebacterium* |
| 4 | *Bacteria* | *Proteobacteria* | *Gammaproteobacteria* | *Pseudomonadales* | *Moraxellaceae* | *Enhydrobacter* |
| 5 | *Bacteria* | *Firmicutes* | *Clostridia* | *Clostridiales* | *Ruminococcaceae* | *Faecalibacterium* |
| 6 | *Bacteria* | *Firmicutes* | *Clostridia* | *Clostridiales* | *Lachnospiraceae* | *Roseburia* |
| 7 | *Bacteria* | *Actinobacteria* | *Actinobacteria* | *Actinomycetales* | *Micrococcaceae* | *Rothia* |
| 8 | *Bacteria* | *Firmicutes* | *Clostridia* | *Clostridiales* | *Clostridiaceae* | *SMB53* |
| 9 | *Bacteria* | *Bacteroidetes* | *Bacteroidia* | *Bacteroidales* | *Bacteroidaceae* | *Bacteroides* |
| 10 | *Bacteria* | *Actinobacteria* | *Coriobacteriia* | *Coriobacteriales* | *Coriobacteriaceae* | *Collinsella* |
| 11 | *Bacteria* | *Firmicutes* | *Erysipelotrichi* | *Erysipelotrichales* | *Erysipelotrichaceae* | *Coprobacillus* |
| 12 | *Bacteria* | *Proteobacteria* | *Gammaproteobacteria* | *Enterobacteriales* | *Enterobacteriaceae* | *-* |
