# Supplementary material for: Bioconversion variation of ginsenoside CK mediated by human gut microbiota from healthy volunteers and colorectal cancer patients
Source: Chin Med. 2021 Mar 17;16:28. doi: 10.1186/s13020-021-00436-z (PMC7968294; doi:10.1186/s13020-021-00436-z)
Supplement: Supplementary file 2 — Additional file 2: Figure S1. Representative MRM chromatograms of blank gut microbiota solution, GCK, PPD and PPT. a1,b1,c1 were the corresponding chromatograms of blank gut microbiota solution; a2: GCK; b2: PPD; c2: PPT. Figure S2. Relative abundance of differentially abundant bacteria in each sample. (a) Bifidobacterium; (b) Blautia; (c) Corynebacterium; (d) Enhydrobacter; (e) Rothia; (f) SMB53; (g) Bacteroides; (h) Collinsella; (i) Coprobacillus; (j) Enterobacteriaceae; significant difference of relative abundance were analyzed using Kruskal-Wallis test and Wilcoxon test; * p < 0.05, ** p < 0.01 and # p < 0.001. Figure S3. Significant functional pathways between health and CRC group by PICRUSt analysis (p < 0.05, LDA scores > 2). [file 13020_2021_436_MOESM2_ESM.pptx]

## Slide 1
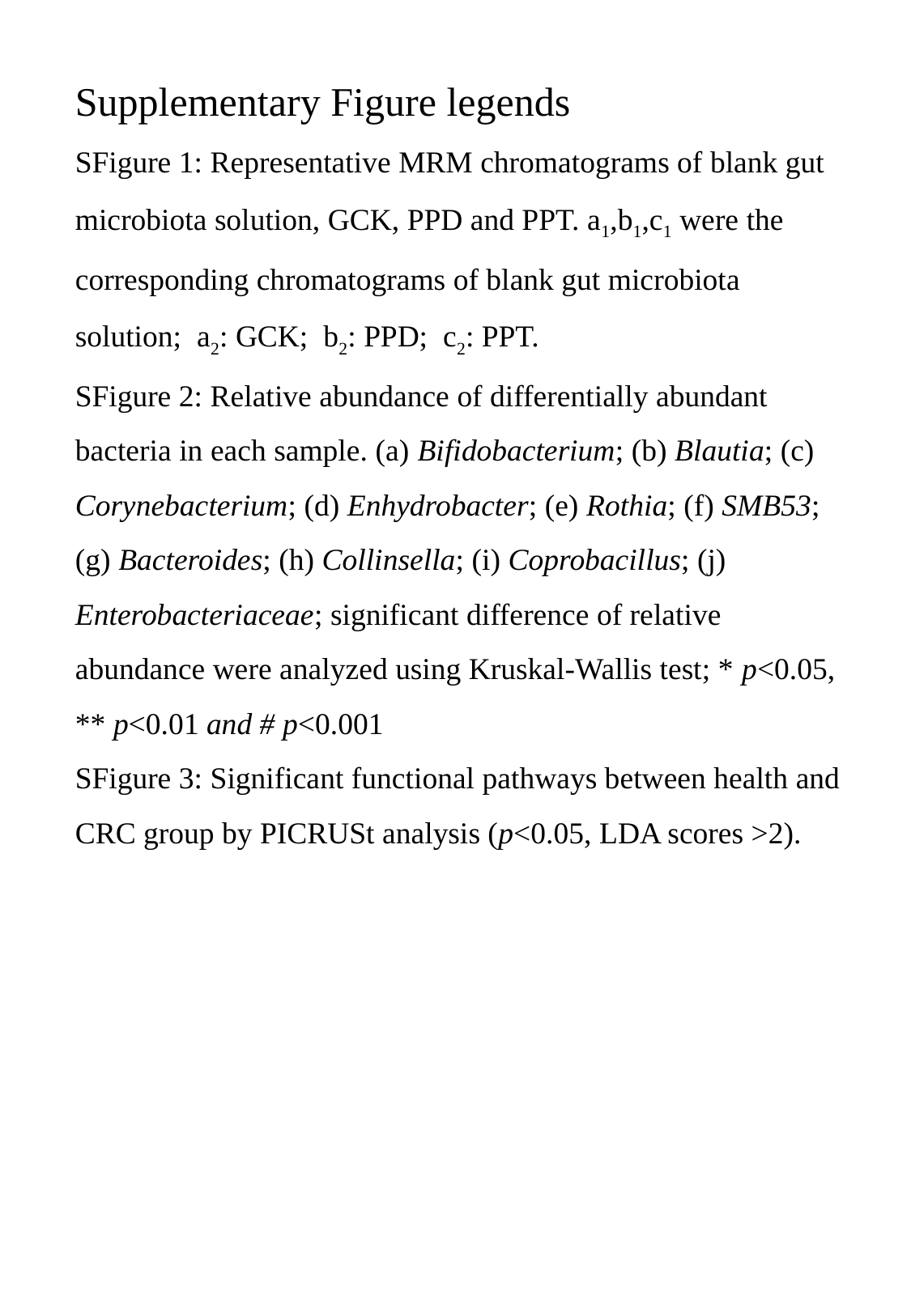

Supplementary Figure legends
SFigure 1: Representative MRM chromatograms of blank gut microbiota solution, GCK, PPD and PPT. a1,b1,c1 were the corresponding chromatograms of blank gut microbiota solution; a2: GCK; b2: PPD; c2: PPT.
SFigure 2: Relative abundance of differentially abundant bacteria in each sample. (a) Bifidobacterium; (b) Blautia; (c) Corynebacterium; (d) Enhydrobacter; (e) Rothia; (f) SMB53; (g) Bacteroides; (h) Collinsella; (i) Coprobacillus; (j) Enterobacteriaceae; significant difference of relative abundance were analyzed using Kruskal-Wallis test; * p<0.05, ** p<0.01 and # p<0.001
SFigure 3: Significant functional pathways between health and CRC group by PICRUSt analysis (p<0.05, LDA scores >2).

## Slide 2
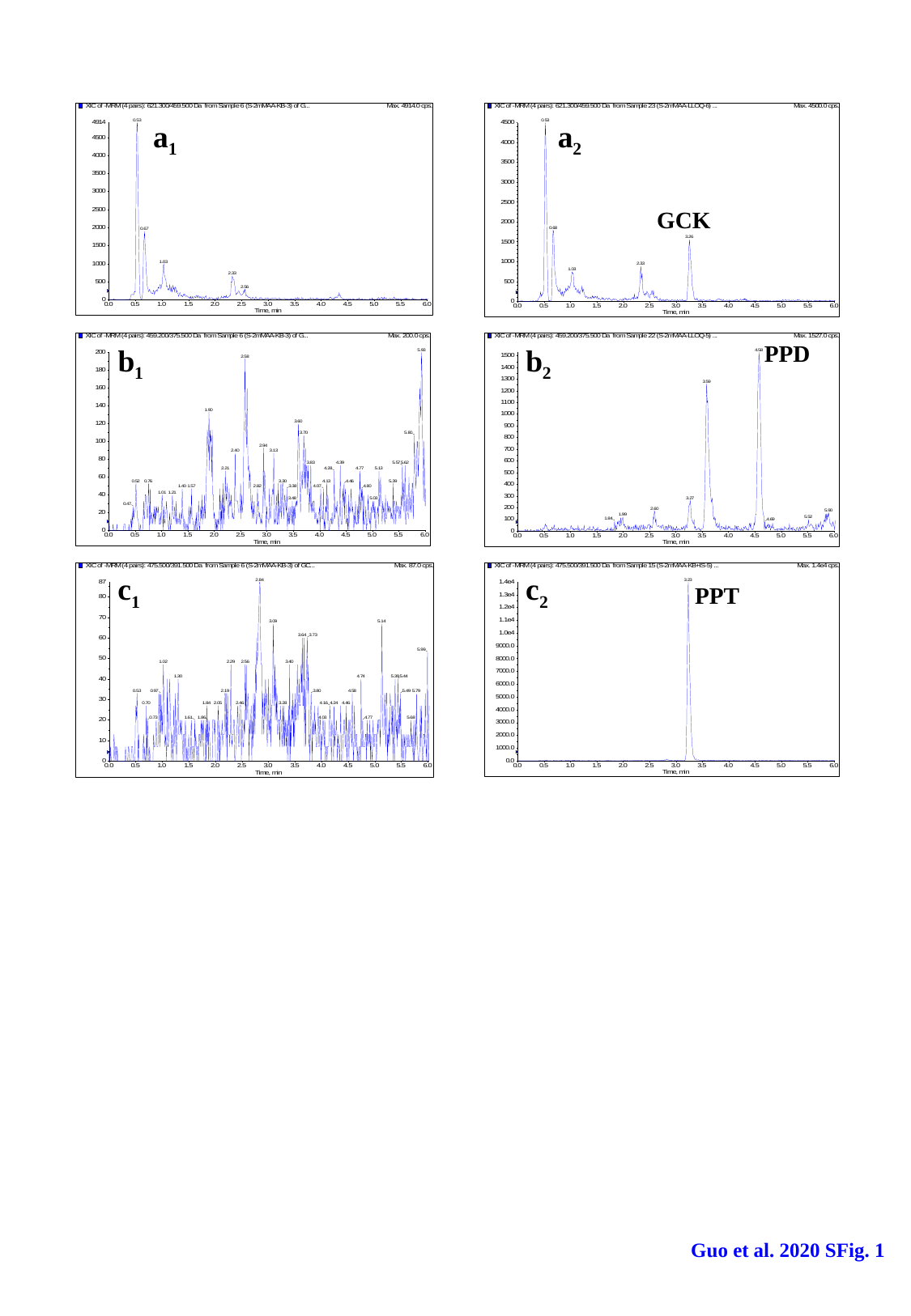

a1
a2
GCK
PPD
b1
b2
c1
c2
PPT
Guo et al. 2020 SFig. 1

## Slide 3
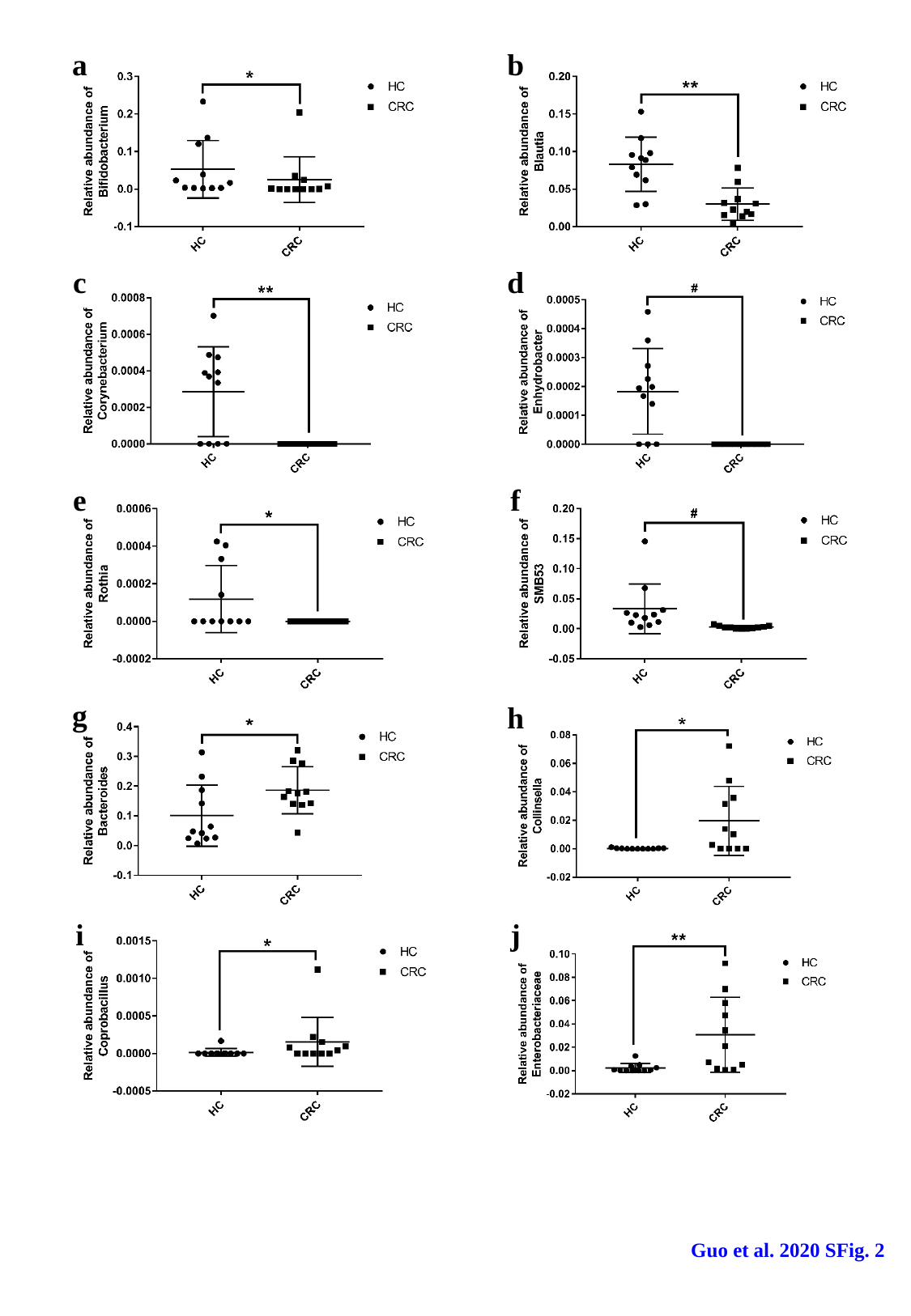

a
b
c
d
e
f
g
h
i
j
Guo et al. 2020 SFig. 2

## Slide 4
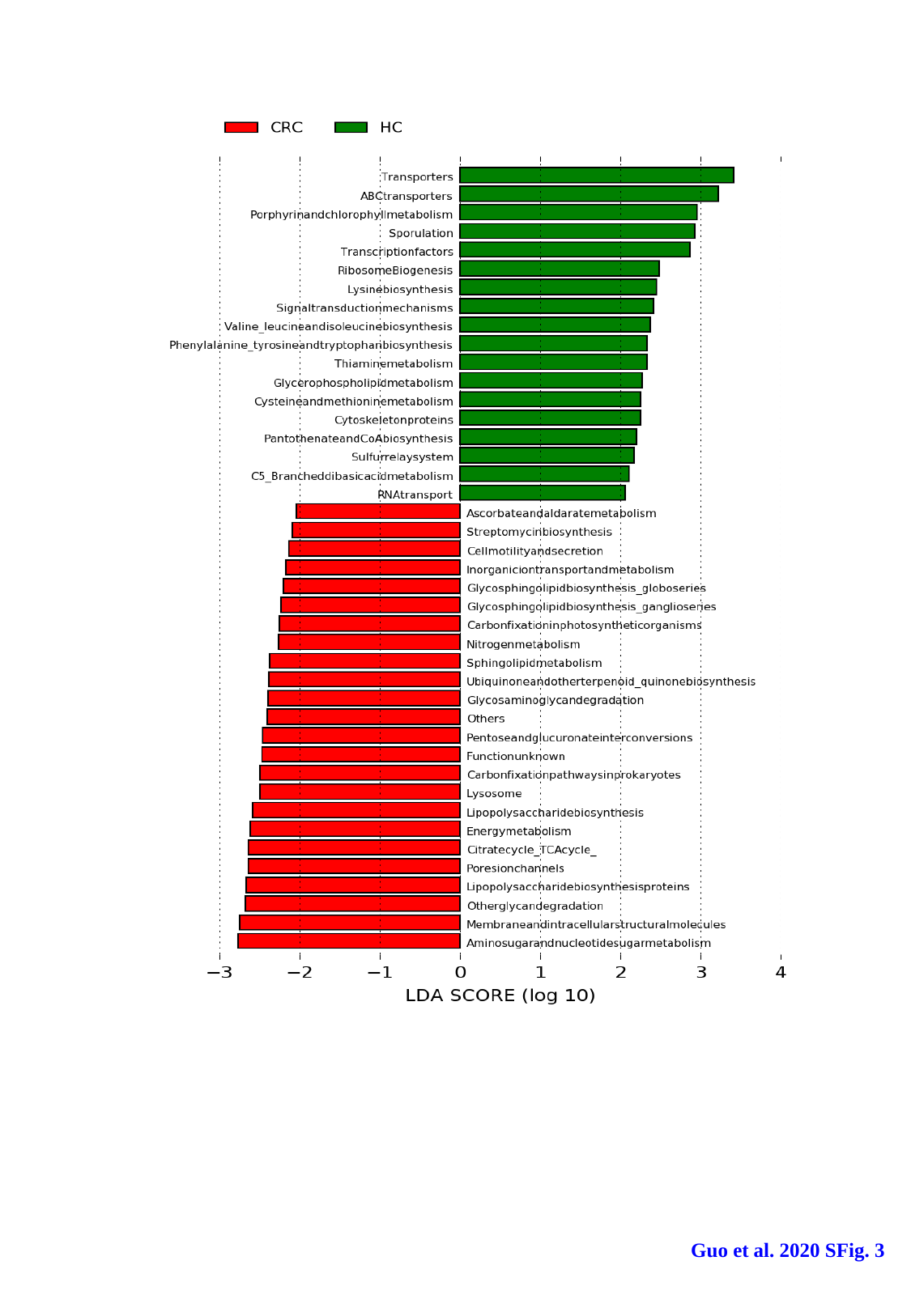

Guo et al. 2020 SFig. 3
